# Daily uplifts during the COVID-19 pandemic: what is considered helpful in everyday life?

**DOI:** 10.1186/s12889-022-12506-4

**Published:** 2022-01-13

**Authors:** Rakel Eklund, Kristina Bondjers, Ida Hensler, Maria Bragesjö, Kerstin Bergh Johannesson, Filip K Arnberg, Josefin Sveen

**Affiliations:** 1 National Centre for Disaster Psychiatry, Department of Neuroscience, Uppsala University, Uppsala, Sweden; 2 Norwegian Centre for Violence and Traumatic Stress Studies, Oslo, Norway; 3 Department of Clinical Neuroscience, Division of Psychology, Karolinska Institute, Stockholm, Sweden

**Keywords:** COVID-19, Daily uplifts, Everyday life, Pandemic

## Abstract

**Background:**

Knowledge of what is uplifting and helpful during pandemics could inform the design of sustainable pandemic recommendations in the future. We have explored individuals’ views on helpful and uplifting aspects of everyday life during the coronavirus disease 2019 (COVID-19) pandemic.

**Methods:**

Participants answered a brief, daily survey via text messages during 14 consecutive days in July–August, 2020. The survey included the question: *“During the past 24 hours, is there anything that has made you feel good or helped you in your life?”* We used content analysis to compile responses from 693 participants, who provided 4,490 free-text answers, which resulted in 24 categories subsumed under 7 themes.

**Results:**

Positive aspects during the COVID-19 pandemic primarily related to social interactions, in real life or digitally, with family, friends and others. Other important aspects concerning work, colleagues and maintaining everyday life routines. One theme concerning vacations, going on excursions and being in nature. Leisure and recreation activities, such as hobbies and physical exercise, also emerged as important, as did health-related factors. Bodily sensations, thoughts, feelings and activities that benefited well-being were mentioned frequently. Lastly, people commented on the government strategies for containing COVID-19, and whether to comply with restrictions.

**Conclusions:**

To summarize, daily uplifts and helpful aspects of everyday life centered around social relationships. To comply with recommendations on physical distancing, people found creative ways to maintain social connections both digitally and face-to-face. Social interaction, maintenance of everyday life routines, hobbies and physical activity appeared to be important for well-being.

## Background

The coronavirus disease 2019 (COVID-19) has created a global health emergency. On the 11th of March 2020, the World Health Organization (WHO) pronounced it a pandemic [1]. In order to slow the transmission of the disease, authorities around the world implemented various measures and restrictions such as closure of schools and limitations of leisure activities and opening hours for restaurants and shops. Such restrictions increase the risks of social isolation, loneliness, financial burdens, and impairment of individuals’ health and well-being [2]. Most people will likely be resilient to the long-term effects of the pandemic with regard to mental health, but some people may experience transient or prolonged increases in psychological symptoms [3–5]. So far, research indicates increased levels of anxiety, depression, and stress during the pandemic [2, 6–10], and that persons forced to isolation due to the pandemic are more likely to develop posttraumatic stress [11]. Thus, long-term management of a pandemic at a societal level requires balancing the physical and mental well-being of populations. Public compliance with regulations requires a sustainable strategy in which individuals are able to cope and maintain an acceptable quality of life, even under difficult circumstances.

Well-being and mental health are affected by major life changes, in addition to minor negative and positive events in daily life, which can be referred to as daily hassles and uplifts [12]. Daily uplifts have been shown to be associated with improved self-assessed health, short-term enhancement of positive affect, work performance, and lesser emotional exhaustion [13–16]. The COVID-19 pandemic and the restrictions deemed necessary to prevent contagion have altered everyday life for most people. A recent study among older adults in the United States found that the most frequently reported sources of joy and comfort were family and friends, digital social contacts and various hobbies [17]. In a global study, which examined coping strategies among participants across 15 nations, the most common coping strategies were watching television, social networking, listening to music, sleeping, doing household chores, eating, and finishing work that had piled up [18]. Another global study investigated both positive and negative impacts of the pandemic, with results indicating that working from home and having less pressure of daily living had a positive impact on everyday life for many people. Strategies reported as helpful were keeping in touch with friends and family, exercising, practicing self-care and being in nature or having access to open spaces [19]. Although these studies provide interesting indications on what people find uplifting, the findings are hampered by the studies’ retrospective cross-sectional designs.

More research into what individuals experience as helpful and positive in daily life during the COVID-19 pandemic is warranted. Systematically collected information about potential uplifts in daily life could provide valuable input into future evidence-based guidelines and advice for maintaining psychological health and well-being during periods characterized by societal restrictions. Therefore, the current study aimed to explore what was helpful and uplifting for Swedes in daily life during the COVID-19 pandemic by using daily assessments during a period of two weeks.

## Methods

### Design

We employed the Intensive Longitudinal Method with a signal-contingent design and a diary component. This means that we prompted the participants to make assessments and obtained repeated and frequent measurements from them, in their natural environment [20].

### Participants, settings and procedure

Participants who had enrolled in the Swedish part of a prospective online survey [21], conducted at Uppsala University, were offered to participate in the current study. Swedish participants were recruited via advertisements in social media from July 7th to August 6th 2020, and asked if they were willing to respond to an online survey consisting of questions regarding psychological health during the COVID-19 pandemic. They were also asked if they were willing to respond to daily measurements via text messages for 14 consecutive days. Inclusion criteria for participating in the study were (1) age 18 years or older (2) could understand Swedish and (3) mainly been in Sweden since the start of the pandemic (March, 2020). The study had no exclusion criteria. Participants received written information about the study and how their data would be processed. They provided written informed consent and entered their contact information into a digital platform. In total, 878 participants consented to participation in the survey, performed daily measurements and provided their contact information. Due to a technical issue, twelve participants were able to register for the study more than once. The aforementioned technical issue also led to four individuals participating during two two-week periods of daily measurements. We excluded two of these participants due to inconsistencies in their responses to the survey. There were 628 participants who provided complete demographic information in the initial survey, whereas 693 participants provided at least one response to the daily question that we analyzed in this study, after removal of uninformative responses that consisted of only a single letter or symbol. We chose to analyze the daily responses also from the 65 participants who failed to complete the initial survey, under the assumption that the distribution of demographic characteristics for these participants did not differ from that of the 628 who did provide demographic information. A majority of the participants (n=628) were female (92.7%), with a mean age of 53 years. The majority had a university degree (76.9%). Half of the participants lived in a large city in Sweden (50.5%) during the time of participation (Table 1).

The following measures were among the societal restrictions applied to prevent contagion in Sweden during the period of data collection: Keep a physical distance to others, work from home, do not travel, use distance learning in higher education, self-isolate when feeling sick. In addition, individuals of more than 70 years of age were asked to maintain strict physical distancing to others and avoid public places such as grocery stores and pharmacies [22, 23]. The compliance with these restrictions was high; in August 2020, approximately 90% of citizens stated that they were complying to a high degree [24].

**Table 1:** Demographic characteristics

Demographic variables	Participants (n=628^a^), *n* (%)
Age (years)	
Mean	53.25
SD	11.14
Median	53
Min–max	20–84
Gender	
Female	582 (92.7)
Male	46 (7.3)
Educational attainment	
University level	483 (76.91)
Below university level	145 (23.09)
Place of residence	
Large city	317 (50.5)
Town	121 (19.3)
Suburb	114 (18.2)
Rural area	76 (12.1)
Income	
1st quartile (0–258,381 SEK)	171 (27.2)
2nd–3rd quartiles (258,282–440,656 SEK)	326 (51.9)
4th quartile (≥ 440,657 SEK)	116 (18.5)
Declined to respond	15 (2.4)

^a^There were 65 participants who did not complete the demographic survey, making the total 628, not 693

### Data collection and material

The data in the current study comprised responses to a free-text item in the daily measurements. Each participants received a daily text message to their mobile phone with a link to a questionnaire that included the following question: *“The outbreak of COVID-19 can affect people in various ways. During the past 24 hours, is there anything that has made you feel good or helped you in your life? If so, please describe.”* The question had a free-text response field and participants could write as much as they liked or nothing at all. The non-nil responses ranged from a single word up to several sentences. In total, 4,490 responses were collected from the 693 participants. Each participant provided a mean of 6.48 (SD = 4.05) free-text responses and the mean word count for the responses was 13 words (median = 8; range = 1 to 965 words). See Table 2 for examples of responses.

**Table 2:** Example of responses and the analysis process

Free-text answer	Codes	Categories	Theme
*Ate a saffron bun, watched a movie, and worked with my art. Little things that make life easier. When life is tough, it feels better if you do nice, little things that make you happy.*	Eat saffron bun	Food & drinks	Leisure activities and recreation
	Watch movie	Hobbies	
	Doing art		
	Nice, little things	Positive experiences	Health and well-being
*Been outdoors with my parents, been with my 77-year-old dad and took a swim in the lake. I am on vacation and I am grateful that it is beautiful weather.*	Be outdoors	Family	Social network
	Socializing with family (Outside)		
	Take a swim	Swim	Leisure activities and recreation
	Vacation	Holidays & vacation	Outings and holidays
	Beautiful weather	Nature	
*I have not read the news and thus nothing about FHM’s (the Public Health Agency of Sweden) statements and therefore I feel more calm and less irritated.*	Do not read the news about COVID-19	COVID-19-related (society)	Being informed and trusting authorities
	More calm, less irritated	Positive experiences	Health and well-being

### Data analysis

**Fig. 1:**
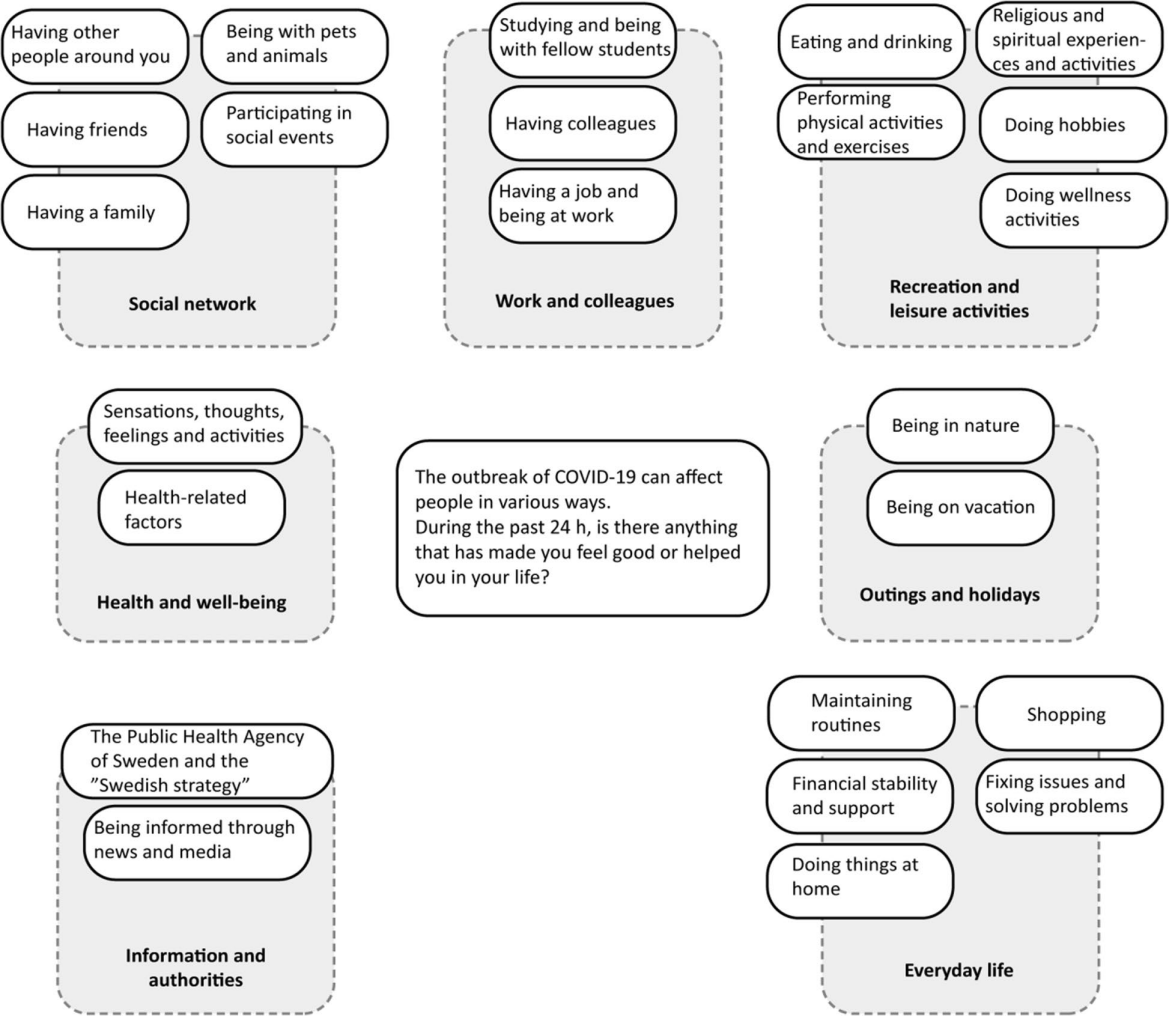
Categories and themes

A conventional content analysis was used [25]. As there was a large number of responses, three authors (RE, JS, FA) started by reading and discussing the contents and possible codes for 100 free-text responses from 22 randomly selected participants. Next, the results from this step were discussed with two other authors (KB, MB) and served as a guide for the subsequent coding process. Five of the authors (RE, JS, FA, KB, MB) then read and coded approximately 900 responses each. The responses were read word by word to derive codes. The five authors who did the analysis discussed their coding during the process in order to remain at the same level of content and prevent any drift in the coding strategy. Each free-text response resulted in between 1 and 10 codes. After the coding step, two of the authors (RE, JS) sorted the codes into categories based on similarity of content. The categories were then sorted into themes by RE and JS; this was followed by discussions among the authors RE, JS, FA, KB and MB (see Table 2 for example from analysis process). The analysis was finished when all of the authors agreed upon the structure and naming of the themes and categories and when illustrative quotations had been selected. The analysis resulted in 24 categories divided across 7 themes (Fig. 1).

## Results

### Social network

 Several kinds of social networks were mentioned by the participants as sources of uplifts. Social groups included family (co-habiting and extended), friends, and others (e.g., neighbors, fellow churchgoers). Having social connections with family and friends was reported as a privilege and in itself serving as a facilitator for well-being. One participant wrote about it as follows: *“Spending time with our children and their partners. Joy and love.”* Having a family provided opportunities for physical closeness (e.g., hugging, kissing, having sex) at a time when the government recommended physical distancing in general. Spending time with pets and animals could provide similar relief through the opportunities to cuddle, as well as to play, go for walks, and watch the pet learn new tasks: *“[I] have a little puppy that keeps me going.”*

Time spent with other people, getting help from or helping others within one’s social network, and making plans for the future, were also described as positive and uplifting. Participants reported that seeing their extended family and friends promoted well-being. Socialization sometimes took place in person and face-to-face (descriptions of this were often worded to highlight social distancing, including phrases like “at a distance” or “outside”): *“Coffee with my husband and my mother, on our terrace, with distance.”* Participants often mentioned specific activities for social interaction, like sharing meals and taking walks. Socialization could also take place remotely via digital platforms, such as video, phone calls, text messages or chat, which served as a means of staying in touch: *“Talked on FaceTime with my son’s family, who called and congratulated me. Talked to mum and dad for a while on the phone.”*

Moreover, helping family and friends (e.g., babysitting, helping out with pets, running errands) was reported as a way to feel needed, which was seen as a source of well-being. Feeling happy over good things happening for a family member or a friend (e.g., when a family member/friend got promoted at work) was also reported to increase well-being. Other things that promoted well-being included future plans to meet other people face-to-face (rather than via digital channels) and plans for participation in social events (e.g., birthdays, graduations, weddings, funerals).

 Further, participants mentioned social groups in settings related to hobbies and faith (e.g., book clubs, church), and chance encounters with neighbors, carpenters and other professionals doing jobs in the home, staff in health care settings or schools, customers, store clerks or fleeting acquaintances. Meeting such people created a sense of connection and fellowship with others, as one participant wrote: *“Being outdoors in the yard, neighbors coming and going, we talk and hang out.”* Participants also mentioned activities such as sharing a meal, having coffee with people, meeting neighbors in the stairwell or having a barbecue in a shared garden. Some participants reported going on dates with new people. Other social events included participating in digital activities, answering comments on social media, or getting help through support groups or phone or digital services.

### Work and colleagues

Work-related facilitators of well-being included both having a job and social relations connected to work: *“travelled to my place of work and hung out with two colleagues.”* Participants described gratitude at having a job and stated that employment created a feeling of security. Getting called for a job interview or being given a new position was reported as positive by some participants.

Both being at work (meeting colleagues, getting away from home) and working from home (safety, calm, physical distancing) were mentioned in positive terms. Some reported feeling good about starting work again after being absent (e.g., when away on sick leave) and enjoyed preparing for future work tasks. One participant wrote as follows: *“Made a big ‘to-do list’ and divided it into smaller sections, so that I can manage better having both a job and studying full-time again when the autumn term starts at the end of August. Stress-reducing.”* The end of the work week or being able to mentally leave work behind during the weekends or during time off were reported as positive experiences.

 Participants described satisfaction from work deriving from feeling competent, using their skills, and feelings of doing something meaningful. Receiving appreciation and positive feedback regarding one’s work was also reported as uplifting: *“I had to focus on solving tricky cases at work that affected other people’s future and lives. I felt that I made a difference and that I was doing good. Got many thanks on Zoom and by e-mail.”*

Meetings with colleagues (face-to-face at work or digitally over the phone or through video-conferencing) were mentioned as facilitators of well-being. Socializing with colleagues created positive interactions and resulted in good conversations and sharing jokes and laughs. Other interactions which the participants related to well-being at work occurred with customers, students, patients, and managers.

Among students, graduation, course examinations and positive feedback regarding one’s school performances were mentioned as facilitators of well-being: *“Positive response from a teacher on the submitted assignment.”* Hanging out with fellow students digitally was also reported as a positive experience.

### Leisure activities and recreation

Many participants mentioned specific hobbies or physical activities which evoked positive feelings (e.g., watching TV or movies, listening to radio or podcasts, creative writing, doing arts and crafts, playing games, looking at funny memes, walking, swimming, indoor cycling, doing martial arts or yoga, horseback riding): *“Running and some physical activity. Then some wine after that and petting the cat.”* Physical or digital visits to cultural institutions, such as museums, botanical gardens, drive-in cinemas, exhibitions, theaters or concerts also facilitated well-being, as one participant wrote: *“I watched a very good theater performance from the Deutsches Theater in Berlin that was available online.”*

Performing physical activities, either outdoors (e.g., walking, running, biking, swimming, kayaking) or indoors (e.g., indoor cycling, aerobics, dancing, martial arts), also evoked well-being, according to some participants. Some traditional indoor activities were performed at home, for instance by following an exercise class via smartphone or TV: “*Workout with Sofia on SVT [Swedish public service television].”*

Moreover, participants reported eating and drinking to be facilitators of well-being. Eating was described in relation to pleasure (good food, good wine), various settings (being able to eat a good breakfast outside, visit a restaurant, café, or pub), a sense of security (having a pantry full of food), sensory experiences (the smell of freshly baked buns), cooking and preparation (making jam, cleaning fish and mushrooms, mixing juices, trying new dishes), future plans (inviting someone over for dinner, preparing a picnic), and as a social activity (e.g., getting berries from a friend, cooking for family and friends). Here, too, responses were often worded using phrases like “outdoors” or “keep a distance”, as seen in the following quote: *“Invited friends over and ate with them. Drank some wine and had a nice time. We were six people, so physical distancing was okay.”*

Various wellness activities, such as taking a bath, putting on make-up, doing spa activities at home or at spa facilities, getting a massage, meditating, practicing mindfulness and doing self-compassion exercises were also reported as promoting well-being.

Some mentioned their faith and belief in God as important for well-being. Activities included participating in religious services (digitally or physically, outdoors), reading the Bible or visiting places of worship.

### Outings and holidays

Planning for, or going on, a vacation was reported as positive for well-being. Participants described activities such as international and domestic travel, taking road trips, going sightseeing, or going on cruises or visits to the archipelago (a classic Swedish holiday destination). Vacations were described as relaxing and de-stressing, but also as sources of gratitude and an opportunity to take care of oneself. Planning for a vacation included booking a trip, packing and looking forward to the trip. Excursions and getting away from everyday life could mean visiting and seeing new places (guided tours, local sightings), as one participant wrote: *“Car trip with my husband in Sweden, with walks, beautiful/exciting places.”*

Being in nature (e.g., in the forest or in the mountains) and partaking of what nature has to offer (e.g., looking at flowers, swimming in lakes and the sea, or going to a beach) were described as everyday life facilitators of well-being during the pandemic: *“enjoy beautiful nature.”* Different activities in nature were mentioned, such as fishing, hiking, picking wild berries and mushrooms, or watching wildlife (e.g., a young seagull or deer): *“walk in the forest, picking blueberries, saw a deer.”* Enjoyment of nature was reported as important for well-being. Examples included enjoying a nice evening and watching the sunset, relishing the chance to breathe fresh air, walking barefoot in the warm summer grass, or lying on the ground watching the clouds. Participants mentioned vacations, excursions, and experiences in nature as activities which promoted well-being in and of themselves, in addition to being social activities that could be shared with family, friends or neighbors.

### Everyday life

Maintaining a routine and structure in everyday life was considered to facilitate well-being. Fixing things and solving problems (e.g., taking the car to the workshop, fixing issues with the computer or fixing the bike), visits to the grocery store or other stores, getting home deliveries, shopping in general or getting help with groceries were reported as activities that increased well-being: *“Nice neighbors who buy groceries for me.”*

Participants’ current homes (e.g., being satisfied with their living situation, having something to come home to) evoked positive feelings, as did the process of moving, getting a new home, or getting a place of one’s own. Doing household chores (e.g., cleaning, refurnishing, renovating, painting) and home-related work outdoors (gardening, chopping firewood, building a new fence or patio) were reported as uplifting. One participant wrote: *“Completed a project: painted the door of the woodwork shed, and started a new project: painting the sauna door.”* Many also reported that well-being derived from simply being in one’s garden, seeing things grow, picking flowers, and being in the sun. Spending time on the balcony or working with potted flowers and plants were also sources of well-being.

Access to a second home, such as a summer home, a country house or a mountain cabin, was reported as a privilege during the pandemic. When getting the chance to visit a second home, participants often used the Swedish expression of “simply being” or “simply existing” (i.e., a feeling of not being rushed, a sense of calm and presence in the moment) and also mentioned working on the house or garden or helping their family members with their summer homes. One participant summed this up as follows: *“Being in our summer home, on the countryside, with my partner and parents-in-law. Here, paradise can remain, despite all the harsh and rough things going on out there.”*

Financial stability and support were other sources of well-being. Examples included having the financial means of buying food, getting new shoes for one’s children, paying bills, getting a higher salary or being granted a loan. Some reported getting financial support from the municipality or the social insurance office as beneficial for well-being.

### Health and well-being

Health during the pandemic was reported as a facilitator for well-being. Being or feeling healthy and strong was described as having a sense of one’s own body strength, being able to move one’s body and happiness at not being severely ill. Participants also described that access to health care promoted well-being, exemplified in getting new medications, treatment or aid for illnesses and getting psychotherapy, supportive talks or rehabilitation: *“Had a good phone call with a nurse regarding my physical health problem, which has been worrying me a lot. Now the anxiety is much less. Glad she called me.”* Specifically related to COVID-19, participants mentioned gratitude and positive feelings about recovering from the virus, being able to socialize more freely after quarantining and recovery, or getting a negative COVID-19 test result.

Aspects related to the health of relatives, such as them feeling better after being ill, their having recovered from COVID-19, or their getting an appointment with a physician or being given treatment were also mentioned as daily uplifts. One participant wrote: *“Everyone around me is pretty healthy, no one close to me has COVID-19 as yet. I feel grateful that I am so privileged.”*

 Participants also mentioned individual sensations, thoughts, feelings and behaviors that increased their feelings of well-being, such as acceptance, gratitude, absence of stress, freedom, self-care, voluntary solitude, focusing on what is good in life, being in the present, noticing positive things or pleasant scents, joy and happiness, hope, expressions of love, laughter, crying, creativity, a feeling of meaningfulness, letting go of worries, seeing opportunities and seizing the day or certain moments. Thoughts, feelings and behaviors could be directed towards oneself, focusing on one’s strengths and knowing that one is loved: *“Focusing on what’s good, on what I can do and what I could influence. Being here and now and taking note of everything positive and everything that I have to be grateful for.”* They could also be directed towards others, e.g., making others happy, feeling kinship, receiving and giving compliments, being able to hug children, and not feeling alone in this: *“that there are two of us, sharing in this.”*

Positive thoughts and feelings related to COVID-19 were also reported, such as feeling hope when death rates were decreasing, or that the time for quarantining would soon be over, that the borders would open again soon and hoping for a vaccine that could being an end to the pandemic.

### Information and authorities

Some participants reported that they found it helpful to watch the regular press conferences held by Swedish authorities. Some described feeling that public health authorities had handled the pandemic properly, that Sweden was doing well compared with other countries, and had positive feelings such as gratitude towards the authorities. One participant compared the response in Sweden to that in France: *“When I think about how good things have been for us in Sweden during the pandemic: that we have avoided a lockdown, that we have not been forced to wear facemasks, and that most people have behaved in a good way, in contrast to how it has been, for example, in France, where we are now.”* Other participants reported that it made them feel good to protest against the public health authorities and their recommendations, as they were not the same in Sweden as in the rest of the world. In Sweden at this time, there was no recommendation on wearing facemasks; some people found it important to discuss this with others, to wear a facemask anyway or to make their own visors.

Some participants described it as helpful to follow the news and media reporting on the pandemic, while others felt it was beneficial to take an occasional time-out: *“Good news (immunity, fewer seriously ill, reduced spread of the virus) affects me”* or *“Not watching the news too much makes me feel better.”* Gaining new knowledge about the virus, learning if and how to use facemasks, getting information about vaccines, and reading good and positive news regarding COVID-19 were all reported as helpful and could give a good feeling to an entire day.

## Discussion

This study examined daily uplifts and helpful aspects of everyday life in the general Swedish population during the first wave of the COVID-19 pandemic. At this time, all citizens were recommended to use physical distancing and individuals over the age of 70 years were asked to maintain strict physical distancing to others. Several themes emerged that appeared to be important during the pandemic. These including staying in contact with friends, families, and others, working, exercising, continuing with hobbies or taking up new ones, being in nature and outdoors, gardening, practicing self-care, maintaining everyday routines such as doing household chores, and eating and drinking well. Positive sensations, thoughts, feelings and activities that benefited well-being were also reported as helpful. Lastly, people mentioned the government strategies for containing COVID-19, and whether or not to comply with restrictions.

In this study, one of the most prominent results of daily uplifts was to have a social network and connections to loved ones. Research suggests that quarantining or social isolation during the COVID-19 pandemic has had a negative impact, increasing people’s feelings of loneliness and affecting their mental health [2, 19, 26]. Modern-day humans often had relatively limited experiences of social isolation, boredom and feelings of loneliness before the COVID-19 pandemic, as we lived in an era of travel and good communication [27]. The findings of our study show that living during a pandemic, with a virus spreading between people, does not necessarily stop people from keeping in touch. Rather, participants found ways – some of them very creative – to keep in contact with their social network, with behaviors and activities that were adapted to the recommendations that applied in Sweden at the time. When participants mentioned face-to-face social contact, it was often described together with words such as “outside” or “at a distance.” Other ways to stay in contact with one’s social network were via telephone, video, chats and other digital means. These results are in line with previous studies, suggesting that staying in contact with family and friends, both face-to-face and digitally, is perceived as a helpful coping strategy [17–19].

Spending time outdoors with others was reported as a daily uplift for many of the participants in this study, which is in line with other studies that have examined the impact on well-being of being outdoors during the pandemic [28, 29]. Being outdoors appeared to facilitate social contact with others, but people also reported appreciation for the outdoors as a space for exercise, resting in a peaceful environment, going on excursions, or doing work in the garden. Similar findings have indicated that being in nature and enjoying the nice weather could elicit joy and be helpful during the COVID-19 pandemic [17, 19], as could gardening, sowing seeds [17, 18, 30] and exercising outdoors [19, 28, 30]. Moreover, similar activities may serve as a means of escape from household chores and confinement [29]. Our results, in conjunction with those of the aforementioned studies, highlight the importance of designing recommendations and restrictions that facilitate citizens’ access to nature or open spaces, to enhance well-being. It should be noted that the survey took place during the summer period in Sweden, which likely enhanced participants’ appreciation of the outdoors and increased their ability to spend time outside. Of course, restrictions cannot depend on the weather, but the results indicate that restrictions from government that facilitate access to nature can promote well-being.

In almost every category identified in this study, maintaining everyday life and routines, upholding life as it was before the pandemic started, and planning for the future, emerged as aspects that the participants reported as lifting their mood. Living during a pandemic, not knowing what future might bring or how long the pandemic might go on, could be a stressful experience, full of uncertainty. In their study, Ivbijaro et al. [19] showed how the disruption of work-life routines and uncertainty about the future regarding personal finances had a negative impact on mental health and well-being during the pandemic. Planning everyday life and sticking to a schedule could be helpful, which both Ivbijaro et al. [19] and the current study showed.

The findings of this study show that the participants engaged in a lot of different hobbies and activities during the pandemic as a source for well-being. Many of these activities or hobbies were forms of creative expression. The finding is coherent with previous research that shows that times of uncertainty, such as living during a pandemic, can increase creativity in people’s everyday life, as a way of coping with the situation [31]. A study performed in France has shown that creativity in everyday life seemed to increase during the COVID-19 lockdown [32].

During the start of the pandemic, Sweden implemented a less restrictive strategy than other countries in Europe. Whereas most countries followed the recommendations from the WHO with closed borders, and firm restrictions on meetings and activities with others through quarantine and lockdowns, Sweden emphasized individual responsibility in complying with recommendations and handling the pandemic, avoiding a general lockdown. During data collection, all citizens were encouraged to exercise physical distancing, especially individuals who were above 70 years of age, who should maintain strict physical distancing to others. The pandemic policy of Sweden has been both criticized and praised during the pandemic and discussions regarding the policy were one finding in this study. Participants in this study followed news reports and listened to updates on how to manage the situation, mainly from the Swedish Public Health Agency. The monitoring behaviors of the participants (following news and reports, listening to and complying with the recommendations from the authorities) were described by them mainly as a means of coping with the situation. However, news and discussions could potentially be experienced as something negative, irritating and frustrating. Indeed, a few participants reported that taking a break from following the news or discussing the governmental response to the pandemic was felt as beneficial, while others enjoyed having discussions on public policy, lockdowns and non-use of facemasks, as well as making comparisons to other countries.

The findings in this study have to be considered with its limitations in mind. This study describes the breadth of uplifts during the pandemic, rather than the frequency or consistency of these uplifts. Therefore, we did not analyze the responses clustered by individuals, which means that the findings do not take into account whether participants gave the same response fourteen days in a row or only responded on one day of the fourteen during data collection. It is also important to know that during the time of data collection (July–August 2020), a large proportion of the Swedish people are on their summer holidays, and that these two months are the two warmest of the year and therefore a much-awaited period. Many people would be outside also in non-pandemic times: passing time in the nature, taking trips, sailing, gardening, or picking berries and mushrooms, which are also reported as results in this study. These results may not be specifically related to the COVID-19 pandemic, as they could be reported as things that Swedes normally do during their summer holidays. On the other hand, the media reported on unusually crowded outdoor recreational areas, such as national parks, and there seems to have been an increase in outdoor activities in Sweden during the pandemic. Furthermore, behaviors occurring at normal times could also be helpful during pandemics.

In the current study, there was an over-representation of females with a university degree and living in a large city, which affects the generalizability of the results. A portion of participants (n = 65) did not provide any demographic information, which further restricts the possibility to draw conclusions on the sample representativity. The large amount of data precluded us from examine if there were possible age or gender differences in activities that were perceived as helpful and uplifting in daily life. In future research, one could use quantitative or mixed methods to explore such differences, to further deepen our understanding of whether certain age groups or gender manage the pandemic period differently.

Strengths of the study are the large sample size and the use of the Intensive Longitudinal Method and content analysis, which have allowed us to bring the research closer to the daily lives of the participants and reduced the risk of retrospective bias compared with a cross-sectional design.

## Conclusions

The knowledge gained from this study about what has been experienced as uplifting and helpful during the pandemic could inform sustainable pandemic recommendations in the future. The results from the current study suggest that despite large disruptions in everyday life, it is possible to create positive experiences for oneself and others, which could make it easier to cope with the difficult circumstances. Public health measures could take this into account by applying recommendations and restrictions that encourage behaviors which could improve mental and physical well-being, while concurrently preventing the spread of disease. Promotion of well-being could be stimulated by informing citizens of potentially helpful activities, or ensuring access to nature and outdoor activities. Also, it seems important to ensure access to open spaces for safely engaging in social, leisure or other well-being activities, particularly for those who do not have access to such spaces themselves or via personal contacts. Providing safe and cheap means of access to recreational areas or providing internet access and digital guidance could enable citizens to stay in touch with their loved ones.

Lastly, we note that even during pandemics and periods of societal crisis, psychological responses are not all negative or all positive. During times of uncertainty, with a potentially life-threatening disease spreading across the world and resulting hassles in everyday life, people still find ways to cope, socialize and feel pleasure, joy, and gratitude.

## Data Availability

The data used and analyzed in this study are available from the corresponding author upon reasonable request.
